# *Bothrops bilineatus*: An Arboreal Pitviper in the Amazon and Atlantic Forest

**DOI:** 10.3389/fimmu.2021.778302

**Published:** 2021-12-15

**Authors:** Paulo Sérgio Bernarde, Manuela Berto Pucca, Ageane Mota-da-Silva, Wirven Lima da Fonseca, Marllus Rafael Negreiros de Almeida, Isadora Sousa de Oliveira, Felipe Augusto Cerni, Felipe Gobbi Grazziotin, Marco A. Sartim, Jacqueline Sachett, Fan Hui Wen, Ana Maria Moura-da-Silva, Wuelton M. Monteiro

**Affiliations:** ^1^ Laboratório de Herpetologia, Universidade Federal do Acre, Cruzeiro do Sul, Brazil; ^2^ Curso de Medicina, Universidade Federal de Roraima, Boa Vista, Brazil; ^3^ Programa de Pós-graduação em Ciências da Saúde, Universidade Federal de Roraima, Boa Vista, Brazil; ^4^ Campus de Cruzeiro do Sul, Instituto Federal do Acre, Cruzeiro do Sul, Brazil; ^5^ Departamento de Ciências Biomoleculares, Faculdade de Ciências Farmacêuticas de Ribeirão Preto, Universidade de São Paulo, Ribeirão Preto, Brazil; ^6^ Laboratório de Coleções Zoológicas, Instituto Butantan, São Paulo, Brazil; ^7^ Departamento de Ensino e Pesquisa, Fundação de Medicina Tropical Dr. Heitor Vieira Dourado, Manaus, Brazil; ^8^ Instituto de Ciências Biológicas, Universidade Federal do Amazonas, Manaus, Brazil; ^9^ Departamento de Pós-Graduação, Universidade Nilton Lins, Manaus, Brazil; ^10^ Escola Superior de Ciências da Saúde, Universidade do Estado do Amazonas, Manaus, Brazil; ^11^ Departamento de Ensino e Pesquisa, Fundação Alfredo da Matta, Manaus, Brazil; ^12^ Núcleo Estratégico de Venenos e Antivenenos, Instituto Butantan, São Paulo, Brazil; ^13^ Laboratório de Imunopatologia, Instituto Butantan, São Paulo, Brazil

**Keywords:** two-striped forest-pitviper, Amazon palm pitviper, snakebite, envenoming, venom, antivenom

## Abstract

The two-striped forest-pitviper (*Bothrops bilineatus*) is an arboreal snake that is currently represented by two subspecies (*B. b. bilineatus* and *B. b. smaragdinus*) that comprise a species complex, and its distribution is in the Amazon and the Atlantic Forest. The rarity of encounters with this snake is reflected in the low occurrence of cases of snakebites throughout its geographic distribution and the resulting low number of published clinical reports. However, in some areas, *B. bilineatus* proves to be more frequent and causes envenomations in a greater proportion. Herein, we review the main aspects of the species complex *B. bilineatus*, including its biology, ecology, taxonomy, morphology, genetic and molecular studies, geographic distribution, conservation status, venom, pathophysiology and clinical aspects, and epidemiology. In addition, the different antivenoms available for the treatment of envenomations caused by *B. bilineatus* are presented along with suggestions for future studies that are needed for a better understanding of the snakebites caused by this snake.

## Natural History of *Bothrops bilineatus*

### *Bothrops bilineatus*: The Snake

**Figure 1:**
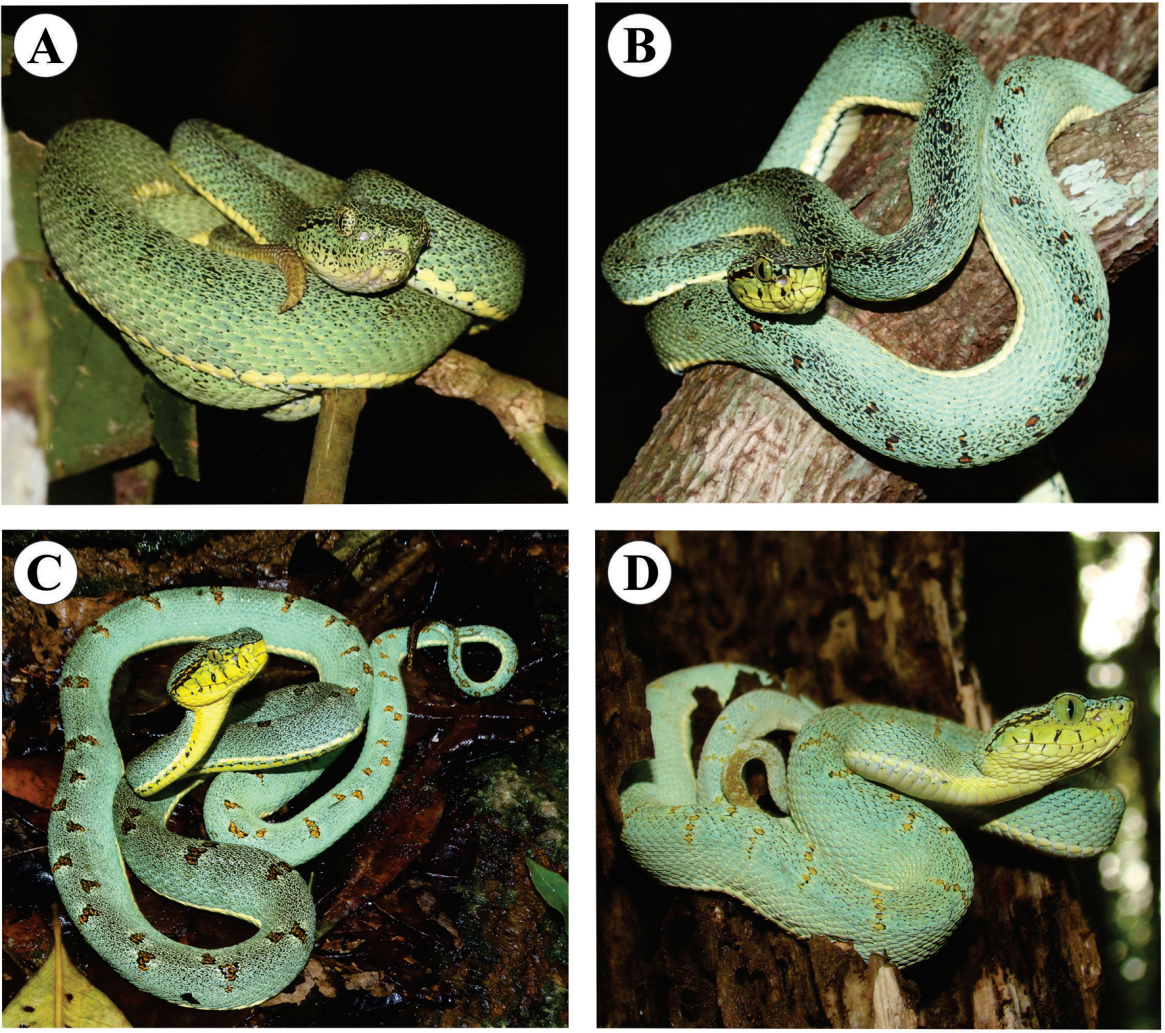
Specimens of *Bothrops bilineatus* from Brazil: **(A)** lower Moa River forest, Cruzeiro do Sul (Acre); **(B)** Tapauá state forest (Amazonas state); **(C)** Murici ecological station (Alagoas state); **(D)** Municipality of Elísio Medrado (Bahia). Photos **(A, B)** by Paulo Bernarde, **(C, D)** by Marco Antonio Freitas.

**Figure 2:**
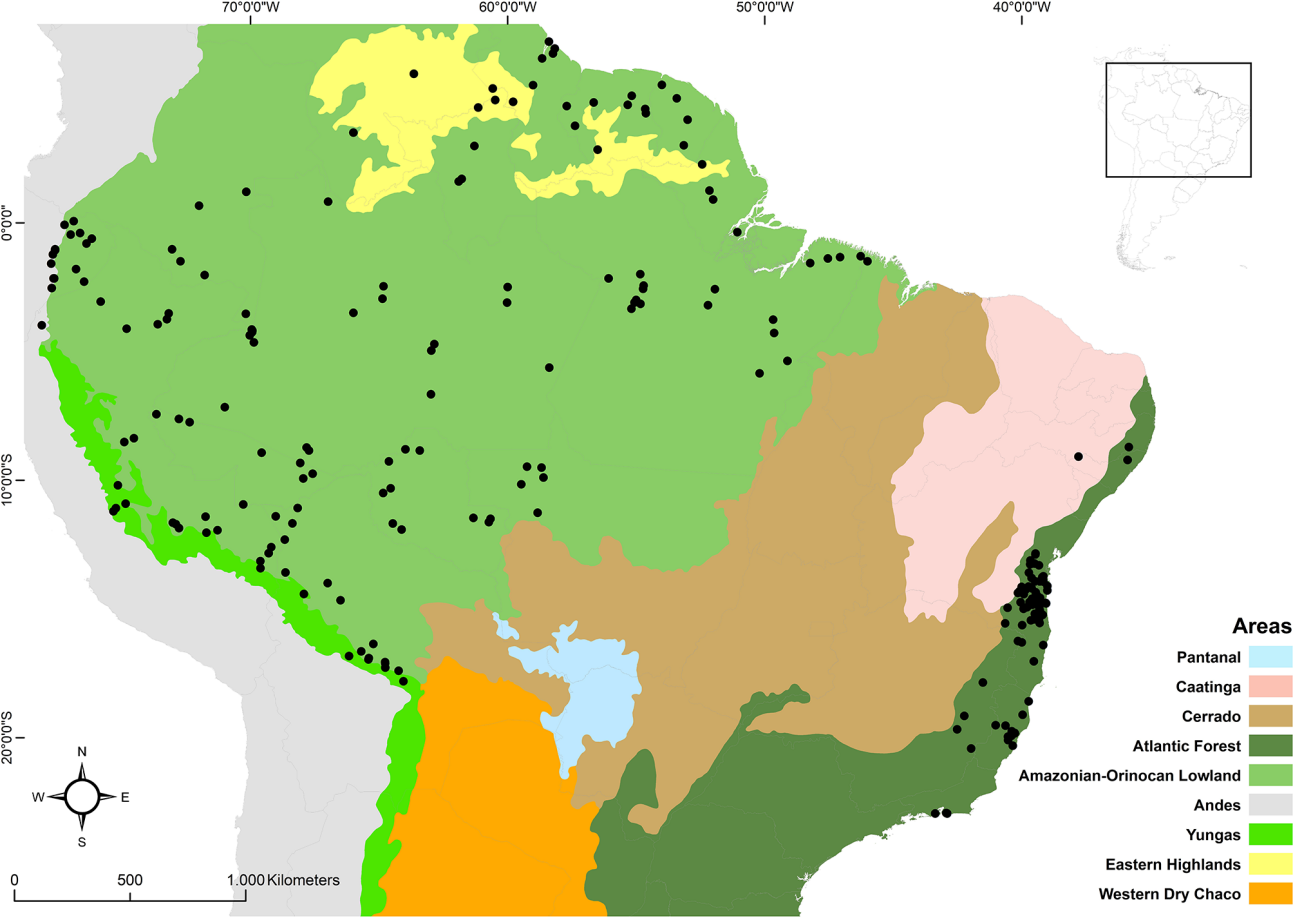
Distribution map of *Bothrops bilineatus* in relation to the ecoregions of South America [Level I, II and III, adapted from Griffith et al., (2)]. See the map with the locations and their bibliographic references in **Supplementary Material**.

The two-striped forest-pitviper (*Bothrops bilineatus*) is an arboreal snake that is currently represented by two subspecies (*B. b. bilineatus* and *B. b. smaragdinus*) that comprise a species complex, and its distribution is in the Amazon and Atlantic Forest, though it is absent in intermediate open and dry habitats (1) (**Figures 1** and **2**). This viperid has adaptations for arboreal life that include a relatively long, prehensile tail and green coloration that makes it hard to spot in vegetation (3). Throughout its geographical distribution it is considered a relatively uncommon snake in studies of snake fauna [e.g. (4, 5)]; however, in the region of Alto Juruá in the state of Acre, it is one of the most common species to be found in some lowland forests (6–8). *Bothrops bilineatus* is also present in cocoa plantations that are contiguous to the forests in Bahia, where it is not considered to be a rare snake in this environment (9). Since it is characteristic of forested environments, its presence does not go unnoticed by residents in the communities, who report the encounters and the snakebites caused by this snake (7, 10–13).

Throughout its geographical distribution, it receives different popular names, which are usually associated with its predominantly greenish coloring and the yellow coloring of the labial region (mainly in the populations of the Atlantic Forest and the eastern Amazon). Known mainly as *jararaca-verde* in the scientific community, it has various regional names in Brazil according to the state in which it occurs, such as *papagaia* (Acre), *bico-de-papagaio* (Mato Grosso and Rondônia), *cobra-papagaio* (Amazonas and Pará), *jararaca-pinta-de-ouro* (Pará), *paraamboia* (Pará), *surucucu-de-patioba* (Pará) and *surucucu-pingo de ouro*, *surucucu-de-ouricana*, *ouricana*, *patioba* (Bahia) (4, 9, 10, 14). In other Amazonian countries, it is known as *víbora-loro* (Bolivia), *loro* and *palo verde* (Ecuador), *loro machaco* (Peru), *mapanare verde* and *mapanare rayada* (Venezuela) (3).

### Taxonomy

*Bothrops bilineatus* has been classified as belonging to the *B. taeniatus* species group by all recent studies that are focused on the systematic of *Bothrops* [e.g. (15, 16)]. The species group is composed of six species of arboreal pit vipers that include *B. bilineatus*, *B. chloromelas*, *B. oligolepis*, *B. medusa*, *B. pulchra*, and *B. taeniatus*. In the last 20 years, the monophyly of the species group has been frequently recovered by molecular phylogenetic analyses (15, 17–24).

However, the relationship between the *B. taeniatus* group and the other species group of *Bothrops* has been a constant matter of debate (15, 21, 22, 24). The contrasting opinions about the classification of this group are reflected in its taxonomic history. The *B. taeniatus* group was considered to be an independent genus, i.e., *Bothriopsis* Peters (1861), for more than 30 years.

Although the species included in the *B. taeniatus* group have been described in several different genera, during the 70’s, all Neotropical diversity of pitvipers was classified as belonging to *Bothrops* [except *Lachesis* ssp.; see Peters and Orejas-Miranda, (25)]. At the end of the 80’s, Campbell and Lamar (26) based on the unpublished dissertation of W.L. Burger (27) split *Bothrops* (*sensu lato*) in five genera: *Bothriechis*, *Bothriopsis*, *Bothrops* (*sensu stricto*), *Ophryacus* and *Porthidium*. From then, until the second decade of this century, all species of the *B. taeniatus* group were referred as belonging to the genus *Bothriopsis*. They were synonymized again with *Bothrops* only after the widespread acceptance of the proposal suggested by Salomão et al. (17), which indicated the nested position of the *B. taeniatus* group within *Bothrops* (15). Therefore, more than 30 years of scientific literature on *B. bilineatus* (and other species of the *B. taeniatus* group) made reference to this species as *Bothriopsis bilineatus*.

*B. bilineatus* was described by Wied-Neuwied (1821) as *Cophias bilineatus*, based on two individuals from Brazil (“Brasilien”). Although undefined in the original description, the type’s location was restricted by Hoge and Lancini (28) to Vila Viçosa (Marobá), Rio Peruhype, Bahia, currently the municipality of Nova Viçosa. Some years later, Hoge (29) evaluated the morphological diversity within the species and described the subspecies, *B. b. smaragdinus*, based on specimens from the region of Alto Rio Purus in the Brazilian state of Acre. In the same study, he suggested the latter was also present in other areas in the western Amazon, in countries like Bolivia, Colombia, Ecuador and Peru.

After the description of *B. b. smaragdinus*, it has been suggested that the nominal subspecies *B. b. bilineatus* is only distributed in two disjunct areas, the northeastern Amazon and the Brazilian Atlantic Forest (1, 3). Although the taxonomic scheme and the geographic understanding of *B. bilineatus* presenting two subspecies is currently accepted, some studies have indicated the possible existence of intermediate morphotypes (30) and distinct genetic lineages (1).

### Morphology

Compared to other species of *Bothrops*, *B. bilineatus* is easily identifiable by its distinctive green color, slender body, and prehensile tail (3). The characteristic pholidosis of the species ranges from 190 to 218 ventral scales in males and from 192 to 220 in females; from 65 to 76 subcaudals in males and 55 to 73 in females; between 23 and 25 scales rows at mid-body; between 5 and 9 intersupraoculars; from 7 to 9 supralabials and 8 to 12 infralabials (3).

The two subspecies of *B*. *bilineatus* differ mainly in their coloration pattern, in which *B*. *b*. *bilineatus* presents vertical dark bars on the supralabials and yellowish or reddish spots on the dorsal region of the body, and *B*. *b*. *smaragdinus* lacks dark vertical bars on the supralabials and the dorsal spots, presenting instead a dorsum punctuated with small black dots (3, 29).

### Genetic and Molecular Studies

In a study based on the phylogeographic analysis of *B. bilineatus*, Dal Vechio et al. (1) show a deep genetic intraspecific structure, which does not exactly mirror the subspecies definition. They recovered a Bayesian phylogenetic tree topology that indicates the presence of four main clades within *B. bilineatus*, thus suggesting that *B. bilineatus* probably represents a complex that includes putative undescribed species. Dal Vechio et al. (1) suggest that these clades are genetic lineages that represent the following geographically restricted populations: 1) Atlantic Forest clade; 2) Guiana Shield clade; 3) Western Amazonian clade; and 4) Central Amazonian clade. The Atlantic Forest and Guiana Shield clades are sister groups, indicating a close relationship between these two lineages. On the other hand, the Western and Central Amazonian clades are relatively more divergent, and have been recovered as successive sister groups of Atlantic Forest and Guiana Shield.

The distribution of the Atlantic Forest and Guiana Shield lineages seems to be easily defined by the limits of the biogeographic regions where they occur. The Atlantic Forest lineage is restricted to the northern portions of the Brazilian Atlantic Forest, occurring in the Brazilian states of Minas Gerais, Espírito Santo, Bahia, Alagoas and Pernambuco (1, 31). While, the distribution of the Guiana Shield lineage seems to be restricted to the Guiana Shield formation, including the territories of Brazil, French Guyana, Guyana, Suriname and probably parts of Venezuela (east of the Orinoco River). In Brazil, this lineage is distributed north of the Amazon River, in the Brazilian states of Amapá, Roraima, northern Amazonas and northern Pará (1, 31).

The Western Amazonian lineage is relatively widespread, occurring in the western regions of the Brazilian Amazon (states of Acre and Rondônia) and in the Amazonian regions of Colombia and Ecuador (probably also occurring in Peru and Bolivia). The eastern limits of the distribution of the Western Amazonian lineage seem to be represented by the Orinoco and Negro Rivers (1). Presenting a similar broad distribution, the Central Amazonian lineage occurs south of the Amazon River, in the Brazilian states of Amazonas, Rondônia, Mato Grosso, Pará (1), and probably also in Maranhão (31).

Although Dal Vechio (2014) (32) in his unpublished Master’s thesis has studied the morphological variability of these genetically defined lineages, he did not formally propose the suggested changes on the taxonomy of *B. b. bilineatus* in any subsequent publication. Based on his results, we can suppose that the Atlantic Forest lineage represents *B. b. bilineatus*, but the allocation of the Guiana Shield lineage into this subspecies or in a new currently undescribed taxon is still open for investigation. The Western Amazonian lineages probably represent the subspecies *B. b. smaragdinus*, and the morphological limits between this lineage and the Central Amazonian lineage seem to be unclear, as well as the limits between the distribution of both lineages. He also suggested that there is evidence to support a possible split of the Western Amazonian lineage into two taxa, one in the north and the other in the south. The Central Amazonian lineage likely represents an undescribed taxon for the *B. bilineatus* complex, which is morphologically and genetically distinct (32).

The taxonomy for *B. bilineatus* will certainly change in the next years. The subspecies will be probably elevated to the species level and at least two new taxa will be described for the complex (32). However, the monophyly of the *B. bilineatus* complex seems to be stable (1), and thus the ecological and toxinological inferences already proposed for *B. bilineatus* will not be affected.

### Size

The maximum sizes reported for *Bothrops bilineatus* in the literature are 1230 mm (33) and 1200 mm (34), for *B. b. smaragdinus* in Venezuela. Harvey et al. (35) recorded 948 mm as the maximum size for *B. b. smaragdinus* in Bolivia. In the Alto Jurá region, west of the Brazilian Amazon, Turci et al. (6) reported two males of 530 (40 g) and 670 (55 g) mm and one female of 780 mm (100 g). Also in Alto Juruá, Fonseca et al. (8) recorded males with 499 to 668 mm (mean 588 mm) and females with 316 to 758 mm (mean 578 mm). In Rondônia, southwest of the Brazilian Amazon, Jorge-da-Silva Jr (36). reported two males (469 to 762 mm) and two females (358 to 825 mm). In Pará, in the eastern Amazon, Cunha and Nascimento (4) and Almeida et al. (37) reported maximum sizes of 822 mm and 980 mm (198 g) for female specimens, respectively. In the Atlantic Forest of Bahia, Argôlo (9) recorded the maximum size of 900 mm for *B. b. bilineatus*. According to literature records, female individuals tend to be larger (3, 6, 8, 36, 37).

The size of young was reported for *B. b. smaragdinus* in Rondônia, with males presenting from 255 to 270 mm (both with 4.1 g) and females with 260 mm (3.7 g), 265 mm (3.7 g) and 265 mm (4.1 g) (38). In Pará, male young with 240 mm (4 g) and 250 mm (5 g) and three females with 232 to 250 mm (4 to 6 g) were recorded (37). Although the smallest individual reported here presented 232 mm, Smalligan et al. (39) reported the shortest length of *B. b. smaragdinus*, which involved a specimen causing envenomation in Peru and which presented 150 mm.

### Geographic Distribution

*Bothrops bilineatus* occurs in lowland rainforests, especially those associated with watercourses (3), and is also present in terra firme and lowland rainforests (4, 5, 8, 36). This snake can also be present in ancient secondary forests near secondary forests (40) and cocoa plantations contiguous to forests (9). Although it is considered infrequent or even rare throughout its geographical distribution (4, 5, 36). In some locations, *B. bilineatus* has been shown to be one of the most abundant snakes (7, 9). In the Atlantic Forest, *B. b. bilineatus* was one of the most abundant snakes in southeastern Bahia (9) and, in Upper Juruá, in a lowland forest, *B. b. smaragdinus* was the most commonly found species during a night search.

In species surveys and snake community ecology studies, *B. bilineatus* is often infrequently recorded, with one to six specimens collected (5, 36, 41–47).

In some locations within its range, this species of snake has not been recorded, denoting that in some regions it is less frequent or even absent (48–50). In the region of Manaus (north of the Amazon River, west of the Negro River, east of the Uatumã River and south of President Figueiredo), despite the snakes sampling efforts carried out by some studies (48, 51–53), *B. bilineatus* was never recorded, and this may reflect a true absence (31).

In some published studies in which snakes were collected in some regions and in which specimen abundance data were provided, *B. bilineatus* corresponded to 0.16 to 28.46% of the specimens in the communities (**Table 1**). Of the 16 studies analyzed, half recorded three or fewer *B. bilineatus* specimens. In four studies in which there were higher numbers of snakes collected (988 to 4680 specimens (9, 36, 40, 57), *B. bilineatus* represented 0.16 to 1.34% of the specimens, denoting its low occurrence in these locations. In only one study carried out in the forest of the lower Moa River (8), *B. bilineatus* was the most abundant snake species and represented 28.46% of the snake specimens recorded in the location. This intriguing greater abundance of *B. bilineatus* in this location of lowland forest when compared to its lower frequency with other studies developed in terra firme areas may indicate that this snake may be less frequent or more difficult to detect by visual searches in other types of vegetation structures (8). In addition, the availability and interactions with their prey should be considered, especially with the abundance and occurrence of *Osteocephalus* amphibians in this location (8).

**Table 1:** Abundance and proportion of *Bothrops bilineatus* in snake communities.

Quantity of specimens of *B. bilineatus*	%	Total snake species	Number of specimens	Location	Reference
1	2.0	25	50	RDS Amanã, Maraã, Amazonas, Brazil	(47)
1	0.56	51	177	RDS Piagaçu-Purus, Anori, Amazonas, Brazil	(54)
1	2.94	23	34	Santo Antônio, Cacoal, Rondônia, Brazil	(55)
1	4.0	18	25	Resex Gregório River, Ipixuna, Amazonas, Brazil	(44)
2	1.96	21	102	Serra do Mandim, Itarantim, Bahia, Brazil	(46)
2	7.40	13	27	Serra da Mocidade, Caracaí, Roraima, Brazil	(56)
2	0.80	70	249	Purus River, Tapauá, Amazonas, Brazil	(45)
2	0.43	51	458	Iquitos, Peru	(41)
3	1.81	47	165	Carajás, Serra Norte, Pará, Brazil	(42)
3	0.60	85	494	Mararu, Santarém, Pará, Brazil	(43)
4	0.39	68	1,016	Samuel Hydroelectric Power Plant, Candeias do Jamari, Rondônia, Brazil	(36)
5	0.16	78	3,118	Northern São Francisco River, Centro de Endemismo Pernambuco, Alagoas, Brazil	(57)
10	6.49	27	154	Vitória, Espírito Santo, Brazil	(58)
11	1.11	88	988	Iquitos, Centro Unión, Peru	(40)
37	28.46	21	130	Forest of the lower Moa River, Cruzeiro do Sul, Acre, Brazil	(8)
63	1.34	61	4,680	Cocoa plantations of southeastern Bahia, Arataca, Bahia, Brazil	(9)

### Habitat, Diel Activity and Abundance

**Figure 3:**
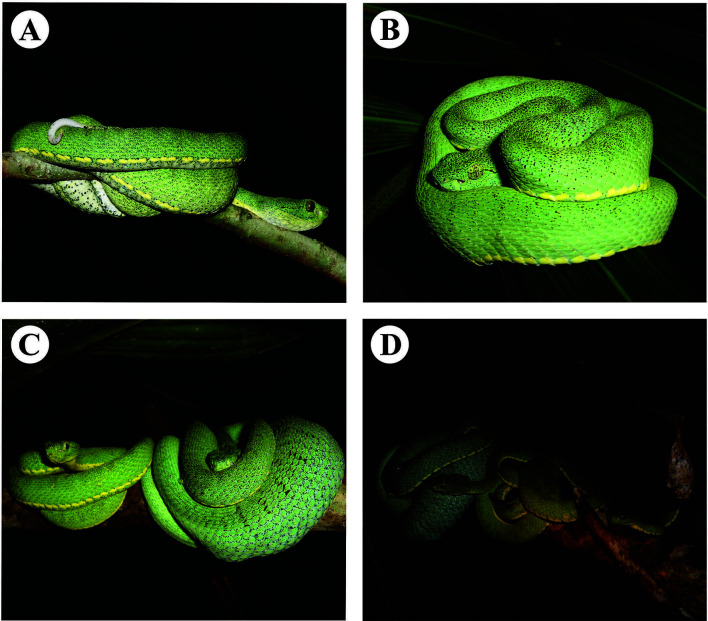
Specimens of *Bothrops bilineatus* in the forest of the lower Moa River (Acre, Brazil): **(A)** specimen performing the caudal decoy tactic; **(B)** specimen resting during the day on a palm frond; **(C)** male individual found together with a female (Note the volume of stomach contents); **(D)** arrival of a second male. Photos by **(A)** Paulo Bernarde, **(B)** Saymon de Albuquerque, and **(C, D)** Luiz Carlos Turci.

Most adult specimens of *Bothrops* species are nocturnal and hunt mainly on the ground and some have a more specialized diet that includes small mammals (e.g., *B. alternatus* and *B. cotiara*), while others are more generalist, feeding on various animal groups (centipedes, anurous amphibians, lizards, other snakes, birds, small mammals) (e.g., *B. atrox* and *B. moojeni*) (59). *Bothrops bilineatus* is a species of nocturnal habits and that hunts predominantly while on vegetation, predating anurous amphibians, lizards, birds, rodents and bats (4, 6, 8, 40, 60–65). Despite it being predominantly nocturnal, Fonseca et al. (8) observed three specimens in standby hunting activity during the early morning hours. During the day, they rest on the vegetation, with the green color providing camouflage among the foliage (**Figure 3B**) (6, 8), usually in the same place that they were hunting at night.

The hunting tactic used by *B*. *bilineatus* is that of waiting, and the snake often uses the caudal decoy behavior (**Figure 3A**), which consists of performing sinuous movements with the tip of the tail that has a distinct color from the rest of the body, thus being able to attract potential prey close to the range of its predatory strike (8, 64). Caudal decoy behavior is performed by juveniles of some species of *Bothrops* (e.g., *B. atrox, B. jararaca, B. jararacussu* and *B. moojeni*) that feed preferentially on anurous amphibians and, during their development, the adult individuals lose this light coloration at the tip of the tail and cease to perform this behavior (59). Adult specimens of *B. bilineatus* retain the distinct coloration of the tip of the tail (white or brown) and continue to use the caudal decoy tactic during hunting activity, probably because ectothermic prey (anurous amphibians and lizards) continue to be important in their diet (59, 64, 66).

Among the species of *Bothrops* that present hunting activity on the vegetation, whether juveniles or eventually adults (e.g., *B. atrox* and *B. jararaca*) and arboreal species (e.g., *B. insularis* and *B. taeniatus*) (3, 59, 67, 68), *B. bilineatus* is the one that has been recorded at higher heights (up to 18 m high) (8). In the state of Alagoas (Atlantic Forest), *B. bilineatus* was observed in hunting activity at 9.4 m, and remained in the same place for three days (69). In a lowland forest in the region of Alto Juruá (State of Acre), western Amazon, *B. bilineatus* was recorded in hunting activity at heights of 30 cm to 18 m (average height of 6.4 m) (8). In the study by Fonseca et al. (8), the authors recorded that individuals of *B. bilineatus* usually stay only one night at the same site (17 observations), with some records of them staying longer; two (9 times) and three (6 times) days, with two specimens that were observed for 15 and 17 days.

### Seasonality

Studies of communities and populations of snakes in the Amazon and the Atlantic Forest have shown that these animals present more activity and are thus more frequent during the wettest and warmest months (5, 48, 70), and more snakebites were also recorded during this period [e.g. (71–73)]. Unlike other snake species, *B. bilineatus* was more frequent during the dry season in a lowland forest in the western Amazon, while its congeneric *B. atrox* was more abundant in the rainy season (7, 8). Fonseca et al. (8) suggested that during the dry season a decrease in humidity occurs in the treetops and some amphibian species (*Osteocephalus leprieuri* and *O. taurinus*) migrate vertically to lower heights in search of more humid environments and *B. bilineatus* would also probably be more active in this forest strata in search of this type of prey.

### Reproduction

*Bothrops bilineatus* is a viviparous snake, with data recording mating between July and August when in captivity, and gestation from 191 to 208 days and parturition in February (74). The number of young in each litter varies from 4 to 15, with a total length of 20 to 27 cm (Starace 1998) (37, 38). Pregnant females in the wild have been collected in September and October and gave birth after about a month in captivity (37, 38). In the wild, Campbell and Lamar (3) reported finding four young in January in Loreto (Peru), and Fonseca et al. (8) observed four young in August (1 young specimen), October (1) and November (2) in Acre (Brazil). Turci et al. (6) observed two males in unsuccessful courting behavior with a resting female (100 g) that had fed on a rodent (60 g) and remained indifferent to the attempts of the males (**Figures 3C, D**). In this observation by Turci et al. (6), a male of smaller size (53 cm of total length and 40 g), moved away from the site with the arrival of another larger male (67 cm; 55 g).

### Conservation

The national Red List of Threatened Fauna categorizes *B. bilineatus* with the status of “Least Concern” (75) and it is not listed on the IUCN Red List (76). However, in the Atlantic Forest, reptile species have suffered a considerable decline, mainly due to the loss and degradation of habitats in this biome and there is still a lack of information regarding the conservation status of the species in several locations (77). In the state of Rio de Janeiro, *B. bilineatus* was considered to be extinct, since it was last recorded in 1963 (73). In the state of the Espírito Santo, *B. bilineatus* was considered vulnerable (78) and, in Minas Gerais, Feio and Caramaschi (79, 80) proposed that this species be included in the Red List of Threatened Species for the state. Throughout its geographical distribution in the Atlantic Forest, it may have had a reduction in its populations and even local extinctions, since it is a forest and arboreal snake, which is probably being harmed by the deforestation that this biome has suffered.

## *Bothrops bilineatus* Snake Venom 

The characterization of the composition of snake venoms is a key step for understanding the evolution and adaptive advantages of each phenotype. It allows us to predict the essential biological events that are triggered by each toxin family and that are responsible for subduing the prey and result in the fitness of the snake species (81). The presence and abundance of different toxins in the venoms also affect the clinical manifestations of patients afflicted by snakebites and indicate the presence of cross-reactive antigens responsible for the efficacy of the antivenom therapy (82). Thus, several studies have attempted to characterize the protein composition of the venoms and their correlation with the mechanisms by which the venoms are capable of inducing the biological effects. Among the main activities evaluated, neurotoxicity, hemostatic disturbances, tissue or cell cytotoxicity, and inflammation are the most studied and use either the whole venoms or isolated toxins. The data relating to *B. bilineatus* venom are highlighted in this section.

Only a few reports on the characterization of *B. bilineatus* venom-derived components are available. However, two recent venomics studies have been published, and contributed with important evidence about the composition of venom from *B. bilineatus* subspecies *bilineatus* and *smaragdinus* (83, 84). The authors analyzed samples of pooled venoms from Peruvian *B. b. smaragdinus* specimens, and also pooled venoms of two *B. b. smaragdinus* specimens originally from Rondonia state, Brazil, and the venom from a single specimen of *B. b. bilineatus* from Rondonia state that had been kept frozen and crystalized for more than 10 years. Interestingly, despite being of different subspecies, geographical origin or conservation conditions, the venom samples exhibited very similar proteomic profiles that comprised components belonging to the most frequent venom protein classes. Snake venom metalloproteinases (SVMPs) were the most abundant toxins with the predominance of the PIII-class; snake venom serine proteinases (SVSP), C-type lectin-like proteins (CTL), phospholipases A2 (PLA2), cysteine-rich secretory proteins (CRISP), and L-amino acid oxidases (LAAO) were also detected in all samples, representing the major components of their venom proteomes. Bradykinin-potentiating-like peptides (BPPs) and the tripeptide inhibitors of SVMPs were also abundant in the three samples analyzed in one of the studies (83, 84). Snake venom vascular endothelial growth factor, nerve growth factor, 5′-nucleotidase, phosphodiesterase, phospholipase B, and others were also detected in minor amounts in venom samples (83, 84). The relative abundance and differences of the major protein families in these venoms are represented in **Table 2**.

**Table 2:** Relative abundance (%) of the major toxin groups in venoms of *Bothrops bilineatus* subspecies.

Subspecies	Protein family (%)								
	SVMP	CTL	CRISP	SVSP	PLA_2_	LAAO	BPPs	SVMPi	Ref.
*bilineatus* (Brazil)	47	10.7	0.9	14.4	3.1	1.3	10.7	8.1	(83)
*smaragdinus (*Brazil)	43.6	10	1.6	7.1	7.6	5	15.3	8.5	(83)
*smaragdinus* (Peru)	58.5	3.2	2.8	5.5	2.8	0.9	14.4	10.1	(83)
*smaragdinus* (Peru)	54.7	15.8	2.6	14.7	1.14	0.28	-	-	(84)

SVMP, snake venom metalloprotease; CTL, C-type lectin-like proteins; CRISP, cysteine-rich secretory proteins; SVSP, snake venom serine proteases; PLA_2_, phospholipases A_2_; LAAO, L-amino acid oxidases; BPP, bradykinin-potentiating-like peptides; SVMPi, snake venom metalloproteases inhibitors.

Considering the most abundant and functionally related proteins, SVMPs comprise a group of zinc-dependent proteases, with a multi-domain organization including catalytic domains bearing the zinc-binding motif and adhesive domains recognized as disintegrin, disintegrin-like, or cysteine-rich domains (85). In the venoms, the two predominant forms are SVMPs from P-I and P-III classes and they are the major toxins in venoms from most species of *Bothrops* snakes (86). Accordingly, in *B. bilineatus* spp, the major toxin group was the SVMPs (83, 84), particularly of the PIII-class (83). These enzymes contain catalytic, disintegrin-like, and cysteine-rich domains. As described for the structurally related toxins present in other *Bothrops* venoms, PIII-class SVMPs are functionally versatile and are involved in most of the local and systemic effects induced by such venoms (87), including the local and systemic hemorrhages (88, 89), inhibition of collagen-induced platelet-aggregation (90–92) and activation of coagulation factors (93–95). SVMPs are also able to induce potent activation of inflammatory mediators (87) and release bioactive cryptides from the extracellular matrix or plasma proteins that act as VAMPs (venom-associated molecular patterns), which activate inflammatory cell Toll-like receptors (96, 97).

SVSPs, are the second most abundant enzymes in *B. bilineatus* venom samples (83, 84). Unlike the SVMPs, most SVSP activities are concentrated on the coagulation dysfunctions induced by *Bothrops* venoms. As reported by Serrano (2013), SVSPs are catalytically active proteins that are able to catalyze the polymerization of fibrinogen into fibrin and are commonly termed thrombin-like enzymes. SVSPs also activate coagulation factors and induce platelet aggregation by cleavage of PAR1-receptors. Anti-coagulant activities of SVSPs have also been described including the activation of plasminogen and protein C, as well as the kallikrein-like activity (98). Disorders of hemostasis are probably enhanced by the presence of considerable amounts of CTLs in *B. bilineatus* samples. CTLs are non-enzymatic components that are functionally relevant in the hemostatic disturbances, which are mostly due to their action on different platelet receptors, and induce aggregation (99) that may lead to the thrombocytopenia observed in some snakebite patients (100). CTLs are also inhibitors of thrombin activity (101, 102).

Different than other venoms of *Bothrops* snakes, the samples of *B. bilineatus* spp venom analyzed by proteomics (83, 84) contained minor amounts of PLA_2_s. PLA_2_s occur in a large variety of venoms, including the venoms of *Bothrops* snakes in basic and acidic isoelectric points (86). Considering the physiopathology that results from *Bothrops* venoms, basic PLA_2_s appear to be the most relevant due to their myotoxicity, while the acidic PLA_2_s are mostly anti-coagulant (103, 104). Interestingly, *B. bilineatus* venom proteomics evidenced low amounts of PLA_2_s, mostly in the acidic form (83, 84).

In addition, although the composition of venoms may vary depending on the region where the snake is found, other studies have shown very similar median lethal dose (LD_50_) values for Brazilian (102.92 μg) and Peruvian (92.74 μg) *B. bilineatus* in mice (18-20 g) (83, 84).

## Envenomation by *Bothrops bilineatus*: Pathophysiology and Clinical Aspects

*Bothrops bilineatus* is classified as Highest Medical Importance (Category 1) in Ecuador, Peru, Colombia and Guyana and of Secondary Medical Importance (Category 2) in Brazil, Bolivia and Venezuela. Although they are highly venomous snakes, exact epidemiological or clinical data are lacking and they are less frequently implicated because of their behavior, habitat preferences, or occurrence in areas remote from large human populations.

The abundance of the above venom-derived toxins in *B. bilineatus* venoms thus explains the signs of envenomations presented by human patients bitten by these snakes, including the local inflammatory reaction, local tissue damage, and coagulation dysfunctions (14, 105, 106). The few reports of snakebites confirmed as being by *B. bilineatus* demonstrate that these envenomations lead to clinical manifestations similar to those caused by other *Bothrops* species, as evidenced in **Table 3**.

**Table 3:** Clinical aspects of envenomations by *Bothrops bilineatus*.

Case	Location	Age (years), sex, occupation	Time until medical care	Local signs	Systemic signs	Treatment	Ref.
1	Itacaré, Bahia, Brazil	26, male, farmer	1 hour and 50 minutes	Intense local pain, burning sensation, “hot” edema at bite site (left index finger) until medium third of left forearm	None	4 vials of *Bothrops* antivenom	(105)
2	Santaré, Pará, Brazil	37, male, unavailable	~30 hours	Erythema, edema, increased local temperature, secondary bacterial infection, blister with serous fluid in the right arm	Acute kidney injury, with oliguria and dark urine, unclottable blood, and prolonged thrombocytopenia	6 vials of *Bothrops* antivenom	(106)
3	Rondônia, Brazil	38, male, farmer	3 days	Immediate intense pain, ecchymosis and edema in the bite site (right scapular region)	Prolonged bleeding from the bite site, gum bleeding, hematuria, and unclottable blood	12 vials of *Bothrops* antivenom	(14)

Regarding the pathophysiological effects resulting from the additive or synergistic action of the toxins, local effects represent a hallmark event during *Bothrops* snakebite. The event consists of the local action of venom toxins that are capable of inducing tissue damage, intense local inflammatory reaction, and muscular impairment, in which SVMPs and PLA_2_ are important components in this sense (107). Associated with the low capacity of antivenom neutralization in the region of the bite, the consequence of local effects can cause longer hospitalization periods and, in some cases, the impairment of limb function (108, 109). After being bitten by *B. bilineatus*, the main local manifestations are characterized by edema, increase local temperature, blister formation and secondary infection (**Table 3**).

During the feeding/capture process, the action of snake venom toxins to block the neuromuscular function of animals is one of the venom strategies to promote prey paralysis and involves myotoxic and neuro-blocking action. In this manner, the group of PLA_2_, found in the venoms as catalytically active or without enzymatic function, represents the main components responsible for these effects (110). The presence of phospholipase activity in *B. bilineatus* venom was reported in early studies conducted by Cadilio and colleagues (1991), though it displays lower activity when compared to other venoms from *Bothrops*, *Crotalus*, and *Micrurus* snakes, which is possibly due to the low amounts observed in *B. bilineatus* venom proteomics (83, 84, 111). Carregari and colleagues isolated the first PLA_2_ from *B. bilineatus* venom, named Bbil-TX, which consists of a calcium-dependent basic PLA_2_ (Asp49) (112). A series of studies have been performed since then in order to understand the action of both crude *B. bilineatus* venom and isolated Bbil-TX on myotoxicity and neuromuscular impairment. From a damage perspective, both venom and toxin were capable of inducing ultrastructural muscular alterations in mouse isolated nerve-phrenic diaphragm preparations *in vitro*, such as hypercontraction of myofilaments, disorganization of sarcomeres, and sarcoplasmic reticulum and mitochondrial damage (113, 114). There are no reports on *in vivo* myotoxicity using crude *B. bilineatus* venom; however, Bbil-TX has induced local myotoxicity in mice after IM administration (represented by increased levels of serum CK peaking at 2 h) with neglected systemic myotoxicity when administrated IV (112). Although the direct myotoxicity to muscle fibers has been reported, studies focused on the neuromuscular impairment caused by *B. bilineatus* venom and Bbil-TX indicate that these alterations were not enough to promote disorder in the contractile mechanism of the muscle. Crude *B. bilineatus* venom and Bbil-TX have also been reported to induce *in vitro* neuromuscular blockade using neuromuscular preparations. Moreover, they did not alter the muscle membrane resting potential or the response of endogenous and exogenous agonists, thus suggesting a presynaptic action and not muscle contraction impairment (113, 115, 116). Moreover, the presynaptic action of Bbil-TX involves modulation of potassium channel activity and presynaptic protein expression, as well as ultrastructural nerve alterations characterized by detachment of the axon from the myelin sheath and formation of periaxonal vacuoles (114, 116).

The inflammatory response involving *Bothrops* venom is an intriguing event during envenomation. The systemic and local immune responses are responsible for triggering several alterations and are considered one of the most important aspects involving local clinical manifestations such as edema and tissue damage. The mechanism involves the direct recognition of venom toxins named VAMPS (117) by immune cells receptors (such as toll-like receptors) and the release of soluble mediators (complement system, cytokines, and chemokines) by the direct action of toxins as well as by the hydrolysis products released from toxins by tissue degradation (extracellular matrix and cell damage products).

*B. bilineatus* venom was found to promote neutrophil migration to the peritoneal cavity, mediated mainly by metalloproteases. Moreover, dexamethasone and zileuton (a 5-lipoxygenase inhibitor) were capable of reducing cell infiltration, indicating that lipid mediators are involved in the venom-induced response (97, 118). As demonstrated for other snake toxins (119–121), the PLA_2_ Bbil-TX was found to induce paw edema and increased serum levels of TNF-α, IL-6, and IL-1 after IM administration (112). In addition to the main pro-inflammatory cytokines studied, Setubal et al. were the first to demonstrate that *B. bilineatus* venom induces IL-8 and PGE_2_ release by neutrophils. In addition, the research group showed that the referred venom stimulates neutrophil extracellular traps (NETs) (122). Indeed, the production of inflammatory mediators, phagocytosis, and the formation of NET_S_ contribute to the clearance of necrotic material, which are important to the reparative and regenerative process after snakebite envenomation. On the other hand, the same components and formation of NETs could be responsible for the pathogenesis of local tissue damage (123). Thus, the role of neutrophils after *B. bilineatus* envenomation needs to be further elucidated.

In regards to systemic effects, *B. bilineatus* venom is capable of promoting hemostatic alterations. This is reported for venoms from other *Bothrops* species and is due to the abundance of SVMPs, SVSPs, and CTLs, which are hemostatically active toxins that are capable of interfering in several events such as the coagulation cascade, platelet function, and fibrinolysis. Moreover, SVMPs are potent hemorrhagins that consist of a class of toxins capable of acting on extravascular targets, such as matrix extracellular components of endothelial basal membranes, in order to induce vessel disruption and consequently local and systemic hemorrhage (124, 125). Rodriguez and colleagues reported that crude *B. bilineatus* venom presented a procoagulant activity in human plasma and fibrinogen; however, it was less potent than that of the Peruvian bushmaster *Lachesis muta* (126, 127). Bilinearin is a 23kDa glycosylated PI-metalloprotease isolated from *B. bilineatus* venom and is capable of inducing prothrombin activation in calcium and phospholipid-independent manner (127). The toxin did not induce isolated fibrinogen clots and was capable to induce human plasma coagulation through prothrombin activation (127). Therefore, the above-mentioned results demonstrate that *B. bilineatus* venom is responsible for a procoagulant activity mediated by the presence of prothrombin activator toxin(s), as well as the presence of thrombin-like toxin(s). As a result, *B. bilineatus* venom was found to promote hemostatic alterations, as cited by Silva (128). The author observed thrombus formation in medium-caliber vessels (such as the pulmonary artery) after histopathological analysis of the lungs of rabbits administered 0.5 mg/Kg of crude *B. bilineatus* venom *via* both IM and IV routes. As a consequence of *Bothrops* envenomations, the venom triggers blood clot formation, and leads to two important possible consequences: (1) the development of an unclottable state, associated with the consumption of hemostatic factors, and culminating in hemorrhagic events; and (2) the formation of intravascular thrombus, resulting in the reduction/cessation of tissue permeability and causing end-organ failure (125). Based on the results of *in vitro* and *in vivo* studies of *B. bilineatus* venom, the procoagulant activity of the venom, mediated by prothrombin activation and thrombin-like activities, is responsible for intravascular coagulation and, consequently, the deposition of thrombus. Although the consumption of coagulation factors has never been reported, it is plausible that the venom interferes in this manner and promotes an unclottable state, which is observed in *B. bilineatus* patients. More clinical and pre-clinical studies on hemostasis involving *B. bilineatus* envenomations are needed to improve our knowledge regarding this important event.

## *Bothrops bilineatus* Snakebites: Epidemiology

Within the geographical distribution of *Bothrops bilineatus*, other species of the genus that are more abundant locally, such as *B. atrox* in the Amazon and *B. jararaca* in the Atlantic Forest, are more common causes of snakebites (3). As such, its rarity or the difficulty in finding this snake in much of its distribution should reflect in the low occurrence of cases of snakebite and also of published clinical reports [e.g. (105, 106)] however, in some locations, *B. bilineatus* is shown to be more frequent: 15% of cases in Letícia (Amazonas, Colombia) (129), 36% in the Pastaza region (Ecuador) (39) and 5.3% in Alto Juruá (Acre, Brazil) (72).

In the Alto Juruá region, *Bothrops bilineatus* was the most commonly found species during night searches, and corresponded to 54% of the snakes, which was more than *B. atrox* (20.7% of the encounters) (7). However, in areas of terra firme in this region, although *B. bilineatus* is present, it is less easy to find and in some studies of species surveys it has not been recorded [e.g. (50, 130)]. Despite its greater abundance in lowland forests in Alto Juruá, snakebites caused by *B. bilineatus* still occur less frequently compared to *B. atrox* (72), which is probably because it is a species that is associated with forested environments and also due to its arboreal habits (8). The average height in which *B. bilineatus* is found in hunting and resting activity is 6.4 m (8), well above the height of a human being, which should contribute to the lower frequency of encounters and snakebites by this snake. Nonetheless, *B. bilineatus* is also found on vegetation at lower heights (less than 2 m in height) and even close to the ground (heights of 30 to 40 cm) (6, 8), which can result in a snakebite when someone accidently bumps into it or approaches it. The occurrence of the bite in the upper regions of the body (fingers of the hand, hand, arm, scapular region, chest and head) is a feature usually associated with envenomation by this snake (13, 105, 106, 129).

Snakebites caused by *B. bilineatus* occur mainly during activities within forests, such as during the opening up of new trails [e.g. (41)] and also extractivism of acai palm (*Euterpe precatoria*) (13). The green coloration of *B. bilineatus* makes it relatively more difficult to spot since it is well camouflaged in this environment (**Figure 3**). When walking in forests, people can bump into this snake on the vegetation and receive a defensive strike, thus envenomation occurs. Wallace (131) mentioned that indigenous people reported that a snake of the genus *Craspedocephalus* (possibly *C. bilineatus*, which was later synonymized with *B. bilineatus*) was very often found on the “piaçaba” (*Leopoldinia piassaba*) and that they were often bitten during the extractivism of this palm. In two cases of snakebite caused by *B. bilineatus* during the acai extractivism, the extractivists were 4 and 8 m high climbing the acai palm and the snake was on a neighboring tree (13). Silva et al. (7) interviewed 100 people who develop some type of activity (hunting, fishing, extractivism) in lowland forests in Alto Juruá and eleven of them reported having already found the “papagaia” (regional, popular name of *B. bilineatus*) on the acai palm or on a neighboring tree. The evidence indicates that *B. bilineatus* usually hunts waiting at the top of the acai trees or in the neighboring trees, waiting for small mammals (rodents and marsupials) that will feed on the fruits of acai, and may eventually bite the extractivists (13).

### Current Treatment of *Bothrops bilineatus* Envenomings

**Figure 4:**
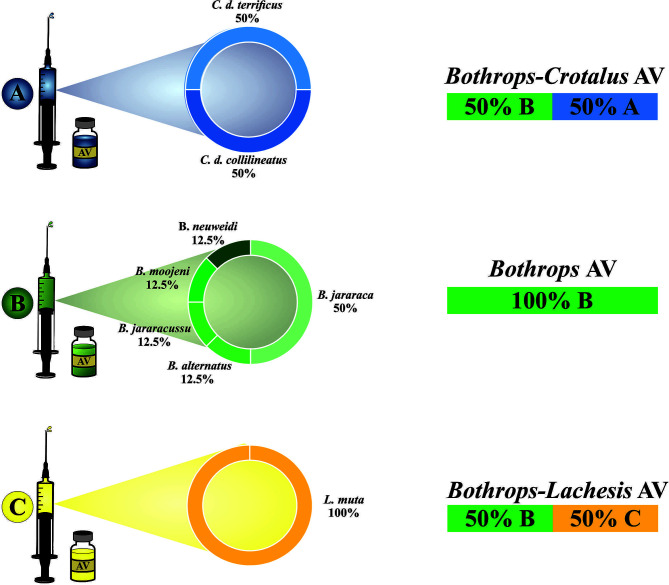
Brazilian antivenoms: venoms used as antigens for horse immunization to obtain specific neutralizing antibodies, and antivenoms currently available in Brazil. These are manufactured from the three different hyperimmune plasma: **(A)** *Bothrops-Crotalus* AV, **(B)** *Bothrops* AV, and **(C)** *Bothrops-Lachesis* AV (right), which can be used to treat *B. bilineatus* envenomations.

The specific treatment for the victims of *B. bilineatus* snakebite is the use of animal-derived antivenoms. So far, there are three different antivenoms (AV) that can be used to treat *B. bilineatus* envenomation: (1) *Bothrops* AV (2) *Bothrops*-*Crotalus* AV and (3) *Bothrops*-*Lachesis* AV (132). Although these three types of antivenoms exist, none are manufactured from the immunization of horses with the venoms of Amazonian *Bothrops* species (**Figure 4** and **Table 4**).

**Table 4:** South American antivenom-manufacturing laboratories and antivenoms manufactured by them.

Laboratory/Country	Antivenom	Venom used in the production process	Neutralizing capacity (mg)/Antivenom (mL)
Instituto Nacional de Laboratorios de Salud/Bolivia	*Bothrops-Crotalus* AV	*B. neuwiedi bolivianus* and *C.d. terrificus*	1.5 mg *B. n. bolivianus* and 0.5 mg *C.d. terrificus*
	*Bothrops-Lachesis* AV	*B. neuwiedi bolivianus* and *Lachesis muta*	2.5 mg *B.n.bolivianus* and 2.5 mg *L. muta*
Instituto Butantan, Instituto Vital Brazil and Fundação Ezequiel Dias/Brazil	*Bothrops* AV	*B. jararaca, B. alternatus, B. jararacuçu, B. moojeni* and *B. neuwiedi*	5 mg *Bothrops* national reference venom (*B. jararaca*)
	*Bothrops-Crotalus* AV	Same for Bothrops AV and *C.d. terrificus*	5 mg *Bothrops* national reference venom (*B. jararaca*) and 1.5 mg *Crotalus* national reference venom
	*Bothrops-Lachesis* AV	Same for *Bothrops* AV and *L. muta*	5 mg *Bothrops* national reference venom (*B. jararaca*) and 3 mg *L. muta*
Instituto Nacional de Salud/Colombia	Polyvalent snake AV	*B. asper, B. atrox*, *C. durissus L. muta* *L. acrochorda* and *Porthidium lansbergii*	7 mg *Bothrops*, 1 mg *Crotalus*, 1.5 mg *L. muta*, 6 mg *L. acrochorda* and 4 mg *P. lanbergii*
Centro Nacional de Productos Biológicos/Peru	*Bothrops* AV	*B. atrox, B. brazili, B. pictus, B. barnetti, and Bothrocophias hyoprora*	2.5 mg *B. atrox*
Centro de Biotecnologia, Facultad de Farmacia de la Universidad Central de Venezuela (BIOTECFAR)/Venezuela	*Bothrops-Crotalus* AV	*Bothrops colombiensis* and *C. d. cumanensis*	2 mg *B. colombiensis* and 1.5 mg *C.d. cumanensis*

A previous study has already shown that *Bothrops* AV, although specific to *Bothrops* genus, demonstrated to recognize and neutralize *Bothrops* venoms from the Amazon Rainforest when compared to *B. jararaca* venom (133), including the venoms of *B. atrox* and *B. bilineatus*. Indeed, an antivenom study demonstrated that the pentavalent antivenom produced by Butantan Institute (*Bothrops* AV) was able to recognize *B. bilineatus* venom by cross-reactivity; however, when *B. b. bilineatus* proteins were analyzed individually, it demonstrated low reactivity for some of them (83).

None of the antivenom-manufacturing laboratories in South America include *B. bilineatus* venom as an antigen (**Table 4**). Clinical studies are scarce and tend to confirm the efficacy of the standard doses recommended in the national guidelines. In one large study performed in the Ecuadorian Amazon, blood coagulation disorders were reverted in almost 68 patients envenomed by *B. bilineatus* (identified by a herpetologist when the snake was brought or by immunoassay determination) within 24 hours after starting treatment with Colombian, Brazilian and Ecuadorian antivenoms (39). Likewise, a case report from the Brazilian Atlantic Forest shows a good clinical response in *B. bilineatus* envenomations when treated with the Brazilian antivenom (105). In contrast, a case reported in the Brazilian Amazon basin revealed a delay in the normalization of the coagulation test, which occurred on day 4-9 of hospitalization, and platelet counts that returned to normal almost 2 weeks after administration of the specific antivenom (106).

The diversity among antivenoms may be evidenced by the different antigens used in the manufacturing process of antivenoms and the values of their neutralizing capacities. As a consequence, treatment schedules vary from country to country. For instance, the Brazilian Ministry of Health establishes 2-4, 4-8 and 12 vials for mild, moderate, and severe *Bothrops* cases, regardless of the species responsible for the envenoming, while the package insert of the Colombian antivenom recommends 2, 4, and 8 vials, according to the severity of the envenomation. Although both liquid antivenoms are supplied in vials of 10 mL, antigens and neutralizing capacities are different, and the ability of these antivenoms to reverse clinical manifestations of *B. bilineatus* envenomations should be confirmed.

Besides the use of specific antivenoms, envenomation caused by *Bothrops* snakes also requires other clinical approaches. Blood pressure monitoring and hydration should be carried out to avoid hypovolemia (134–136). For the management of compartmental syndrome, the conservative attitude is the best approach, and fasciotomy should be indicated with caution (137). Surgical debridement of necrotic tissue in the affected limb may require long-term follow-up to avoid functional or permanent disability.

The use of broad-spectrum antibiotics, such as ampicillin, ceftriaxone, ciprofloxacin, and clindamycin, should be considered, as well as tetanus prevention measures, except for the administration of the tetanus vaccine, which should be avoided within 48 hours after the bite and/or if coagulopathies persist (136). To avoid acute kidney injury (AKI), the use of vasoactive and diuretic substances must be judicious, as well as hydroelectrolytic correction (134, 138).

## Study Perspectives

Future molecular and taxonomic studies should reveal the status of the species within the complex *Bothrops bilineatus*, which will be the basis for research on the variation of toxins and for the comparison of interspecific bioecology. Regarding bioecology, further research is needed to better elucidate the abundance, seasonal patterns and living area of *B. bilineatus* in the locations where it is most frequent, a lowland forest in the western Brazilian Amazon.

Some species of *Bothrops* that present ontogenetic changes in the diet, whose juveniles predate mainly ectothermic prey (e.g., anurous amphibians and lizards) and endothermic adults (e.g., rodents), are characterized by differences in the composition of their toxins that are related to the animal groups on which they feed. The hunting tactic of caudal decoy to attract amphibians that is widely used also by adult individuals instigates the conduct of studies on possible ontogenetic variation of toxins.

The scarcity of clinical reports of poisoning by *B. bilineatus* makes the knowledge about the therapy relatively incipient, which when associated with the peculiar epidemiological circumstances of the snakebite usually involving higher regions of the body and associated with human activities within forests, provides the need to expand the clinical and epidemiological studies with this snake.

## Final Remarks

The snake *Bothrops bilineatus* is a species complex encountered in forests of the Amazon and the Atlantic forest, which presents arboreal habits and different population densities throughout its geographical distribution, being generally uncommon in some areas and relatively more abundant in others. These differences in the abundance of *B. bilineatus* probably reflect in the proportion of cases of snakebites in each region and their rarity in much of its geographical distribution.

The arboreal and forest habits of *B. bilineatus*, usually occurring at heights greater than the height of a human being, tends to link the snakebites with human activities within the forest (hunting, fishing) when it is on the lower strata of the vegetation or when the person is climbing palm trees during extractivism. Its green coloration and sedentary behavior make *B. bilineatus* well camouflaged in the environment, which increases the chances of it not being seen and, when approached or touched by a human, its defensive response often ends in an envenomation. Since extractivism often requires climbing palm trees and other trees, care and attention are essential in order to prevent envenomations by this snake.

## Author Contributions

PS and WM conceived the main idea of this work. All authors designed and wrote most of this review’s topics. FC, MA, and WF elaborated the figures of this review article. All authors corrected the manuscript and provided important contributions during the development of this work. All authors contributed to the article and approved the submitted version.

## Funding

We thank *Conselho Nacional de Desenvolvimento Científico e* Tecnológico (CNPq, The National Council for Scientific and Technological Development, scholarship to MP no. 307184/2020-0, WM n. 309207/2020-7, to AMM-d-S no. 303958/2018-9, and PS 311509/2020-7) and *Fundação de Amparo à Pesquisa do Estado de São Paulo* (FAPESP, São Paulo Research Foundation; research grant 2016/50127-5; scholarship to IO no. 2020/13176-3). WM acknowledges funding support from *Fundação de Amparo à Pesquisa do Estado do Amazonas* (PAPAC ¸ 005/2019, PRO-ESTADO and Posgrad calls). MP (Snakebite Roraima project coordinator) acknowledges funding support from Hamish Ogston Foundation - Global Snakebite Initiative.

## Conflict of Interest

The authors declare that the research was conducted in the absence of any commercial or financial relationships that could be construed as a potential conflict of interest.

## Publisher’s Note

All claims expressed in this article are solely those of the authors and do not necessarily represent those of their affiliated organizations, or those of the publisher, the editors and the reviewers. Any product that may be evaluated in this article, or claim that may be made by its manufacturer, is not guaranteed or endorsed by the publisher.
