# Supplementary material for: Bothrops bilineatus: An Arboreal Pitviper in the Amazon and Atlantic Forest
Source: Front Immunol. 2021 Dec 15;12:778302. doi: 10.3389/fimmu.2021.778302 (PMC8714932; doi:10.3389/fimmu.2021.778302)
Supplement: Supplementary file 1 [file DataSheet_1.docx]

Supplementary Material

# Supplementary Figure


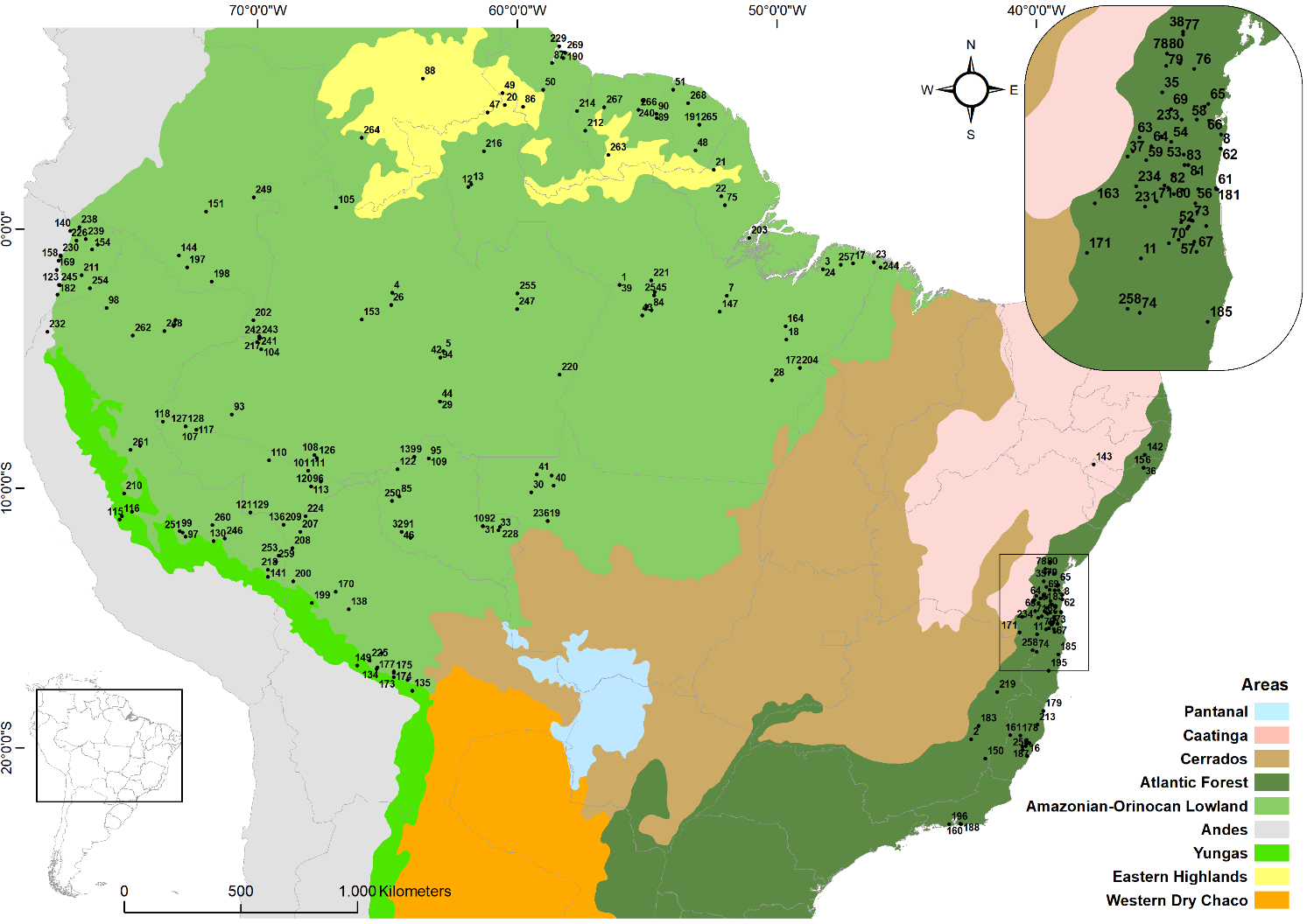


**Supplementary Figure 1.** 1- Juruti, Santarém, Pará, BR (1); 2- Parque Estadual do Rio Doce Marliéria, Minas Gerais, BR (2); 3- Matas do retiro Santa Barbara, Benevides, Pará, BR (3); 4- RDS Amanã, Maraã, Amazonas, BR (4); 5- RDS Piagaçu-Purus, Baixo Rio Purus, Amazonas, BR (5); 6- ESEC Murici, Murici, Alagoas, BR (6); 7- Senador Jose Porfirio, Pará, BR (7); 8- Itacaré, Bahia, BR (8); 9- Porto Velho, Rondônia, BR (9); 10- Cacoal, Rondônia, BR (10); 11- Serra do Mandim, Itarantim, Bahia, BR (11); 12- Serra da Mocidade, Caracaí, Roraima, BR (12); 13- Serra da Mocidade, Caracaí, Roraima, BR (12); 14- Mararu, Santarém, Pará, BR (13); 15- Norte Rio São Francisco, Alagoas, BR (14); 16- Vitória, Espírito Santo, BR (15); 17- Ourém, Pará, BR (16); 18- Tucuruí, Pará, BR (17); 19- Mato Grosso, BR (17); 20- Uiramutã, Roraima, BR (17); 21-PARNA Montanhas do Tumucumaque, Amapá, BR (17); 22-Serra do Navio, Amapá, BR (17); 23- Viseu, Bela Vista, Pará, BR (17); 24- Matas do retiro Santa Barbara, Benevides, Pará, BR (17); 25- Mararu, Santarém, Pará, BR (17); 26- RDS Mamirauá, Amapá, BR (17); 27- Curuá-Uma, Pará, BR (17); 28- Serra Norte, Carajás, Pará, BR (17); 29- Floresta Estadual do Tapauá, Amapá, BR (17); 30- Fazenda Maracatiá, Aripuanã, Mato Grosso, BR (17); 31- Cacoal, Rondonia, BR (17); 32- PES Guajará-Mirim, Rondônia, BR (17); 33- Fazenda Jaburí, Espigão do Oeste, Rondônia, BR (17); 34- Olivença, Bahia, BR (18); 35- Wenceslau Guimarães, Bahia, BR (18); 36- ESEC Murici, Murici, Alagoas, BR (18); 37- Boa nova, Bahia, BR (18); 38- Serra da Jiboia, Varzedo, Bahia, BR (18); 39- Juruti, Pará, BR (18); 40- Cotriguaçu, Mato Grosso, BR (18); 41- Colniza, Mato Grosso, BR (18); 42- Rio Purus, Amazonas, BR (18); 43- Tapajós, Pará, BR (18); 44- Floresta Estadual do Tapauá, Amazonas, BR (18); 45- Santarém, PA, BR (18); 46- PES Guajará-Mirim, Rondônia, BR (18); 47- Pacaraima, Roraima, BR (18); 48- Guiana Francesa (18); 49- Maringma-tepui, Guiana (18); 50- Potaro-Siparuni, Guiana (18); 51- Petit Saut, Guiana Francesa (18); 52- Cacauais do Sudeste da Bahia, Arataca, Bahia, BR (19); 53- Cacauais do Sudeste da Bahia, Aurelino Leal, Bahia, BR (19); 54- Cacauais do Sudeste da Bahia, Barra do Rocha, Bahia, BR (19); 55- Cacauais do Sudeste da Bahia, Barro Preto, Bahia, BR (19); 56- Cacauais do Sudeste da Bahia, Buerarema, Bahia, BR (19); 57- Cacauais do Sudeste da Bahia, Camacan, Bahia, BR (19); 58- Cacauais do Sudeste da Bahia, Camamu, Bahia, BR (19); 59- Cacauais do Sudeste da Bahia, Dário Meira, Bahia, BR (19); 60- Cacauais do Sudeste da Bahia, Ibicaraí, Bahia, BR (19); 61- Cacauais do Sudeste da Bahia, Ilhéus, Bahia, BR (19); 62- Cacauais do Sudeste da Bahia, Itacaré, Bahia, BR (19); 63- Cacauais do Sudeste da Bahia, Itagi, Bahia, BR (19); 64- Cacauais do Sudeste da Bahia, Itagibá, Bahia, BR (19); 65- Cacauais do Sudeste da Bahia, Ituberá, Bahia, BR (19); 66- Cacauais do Sudeste da Bahia, Maraú, Bahia, BR (19); 67- Cacauais do Sudeste da Bahia, Mascote, Bahia, BR (19); 68- Cacauais do Sudeste da Bahia, Mutuípe, Bahia, BR (19); 69- Cacauais do Sudeste da Bahia, Nova Ibiá, Bagia, BR (19); 70- Cacauais do Sudeste da Bahia, Pau Brasil, Bahia, BR (19); 71 Cacauais do Sudeste da Bahia, Santa Cruz da Vitória, Bahia, BR (19); 72- Cacauais do Sudeste da Bahia, Uruçuca, Bahia, BR (19); 73- PARNA Serra da Lontra, Arataca, Bahia, BR (20); 74- Fazenda Alto Cariri, Salto da Divisa, Minas gerais, BR (21); 75- Serra do Navio, Parque Natural Municipal do Cancão, Amapa, BR (22); 76- Serra da Jiboia, Valença, Bahia, BR (23); 77- Serra da Jiboia, Elíseo Medrado, Bahia, BR (23); 78- Serra da Jiboia, Amargosa, Bahia, BR (23); 79- Serra do Timbó, Ubaíra, Bahia, BR (24); 80- Serra do Timbó, Amargosa, Bahia, BR (24); 81- Bahia (25); 82- Lagoa Encantada e Rio Almada, Serra dos setes paus, Bahia, BR (26); 83- APA Lagoa Encantada e Rio Almada, Serra da Temerosa, Bahia, BR (26); 84-Rio Curuá-Uma, Agropecuária Treviso, Pará, BR (27); 85- PES Guajará-Mirim, Rondônia (27); 86- Paramakatoi, Guiana (28); 87- Near Kartabo, Guiana (29); 88- Guiana Venezuelana, Venezuela (30); 89- Lely Mountains, Suriname (31); 90- Kwamalasamutu, Sipaliwini, Suiriname (32); 91- Guajará-Mirim, Rondônia, BR (33); 92- Cacoal, Rondônia, BR (33); 93- Reserva Extrativista do Rio Gregório, Amazonas, BR (34); 94- Rio Purus, Tapauá, Amazonas, BR (35); 95- Samuel Hydroelectric Power Plant, Candeias do Jamari, Rondônia, BR (36); 96- Rio Branco, Acre, BR (37); 97- Cashiriari, Cusco, Peru (38); 98- Andoas, Loreto Derpartment, Peru (38); 99- Malvinas, Cusco, Peru (38); 100- Porto Acre, AC, BR (37); 101 Bujari, Acre, BR (37); 102- Floresta do Baixo Rio Moa, Cruzeiro do Sul, AC, BR (39); 103- Usina Hidrelétrica Santo Antônio, Porto Velho, Rondônia, BR (40); 104- Benjamin Constant, Amazonas, BR (17); 105- São Gabriel da Cachoeira, Amazonas, BR (17); 106- Floresta do Baixo Rio Moa, Cruzeiro do Sul, AC, BR (17); 107- Cruzeiro do Sul, Acre, BR (17); 108- Alto Purus, Amazonas, BR (17);109- Samuel Hydroelectric Power Plant, Rondonia, BR (17); 110- Alto Purus, Acre, BR (17); 111- Bujari, Acre, BR (17); 112- Porto Acre, AC, BR (17); 113- Rio Branco, AC, BR (17); 114- Pichanaki, Peru (9); 115- Vitoc, Peru (9); 116- Chanchamayo, Peru (9); 117- BR-364, Cruzeiro do Sul, AC, BR (18); 118- PARNA Serra do Divisor, Mâncio Lima, AC (18); 119- Usina Hidrelétrica Santo Antônio, Porto Velho, Rondônia, BR (18); 120- Rio Branco, Acre, BR (18); 121- ESEC Rio Acre, Assis BR (18); 122- Usina Hidrelétrica Jirau, Porto Velho, Rondônia, BR (18); 123- Morona Santiago, Macuma, Equador (18); 124- Leticia, Amazonas, BR (18); 125- Sucumbios, Equador (18); 126- Alto Purus, Amazonas, BR (41); 127- Floresta do Baixo Rio Moa, Cruzeiro do Sul, AC, BR (42); 128- Floresta do Baixo Rio Moa, Cruzeiro do Sul, AC, BR (43); 129- ESEC Rio Acre, Assis Brasil, AC (44); 130- PARNA Manu, Madre de Dios, Peru (45); 131- Reserva Florestal Santa Cruz, Peru (46); 132- Estación de Biodiversidad Tiputini, Equador (47); 133- Explorer’s Inn, Peru (48); 134- Alto Chipiriri, Cochabamba, Bolívia (49); 135- PARNA Amboró, Bolívia (49); 136- Nicolás Suárez, Pando, Bolívia (49); 137- Ichilo, Departamento Santa Cruz, Bolívia (49); 138- Estacion Biosferica del Beni, Bolívia (49); 139- Porto Velho, Rondônia, BR (33); 140- Yachana Reserve, Boca Dashiño, Equador (50); 141- La Nube Biological Station, Bahuaja-Sonene National Park, Peru (51); 142- RPPN Frei Caneca (52); 143- Norte Rio São Franscico, Alagoas (14); 144- La Chorrera, Colômbia (53); 145- Boa nova, Bahia, BR (53); 146- Almadina, Bahia, BR (53); 147- Altamira, Pará, BR (53); 148- Coaraci, Bahia, BR (53); 149- Alto Chipiriri, Cochabamba, Bolívia (53); 150-Alto Jequitibá, Minas Gerais, BR (53); 151- Alto Rio Apaporis, Vaupés, Colômbia (53); 152- Arataca, Bahia, BR (53); 153- Caitau, Amazonas, BR (54); 154- Cononaco, Equador (53); 155- Ilhéus, Bahia, BR (53); 156- Aracruz, Espírito Santo, BR (53); 157- Aracruz, Espírito Santo, BR (53); 158- Arajuno, Equador (53); 159- Arataca, Bahia, BR (53); 160- Maricá, Rio de Janeiro, BR (53); 161- Baixo Gandu, Espírito Santo, BR (53); 162- Santarém, Pará, BR (53); 163- Barra do Choça, Bahia, BR (53); 164- Tucuruí, Pará, BR (53); 165- Benjamin Constant, Amazonas, BR (53); 166- Brownsberg, Suriname (53); 167- Buerarema, Bahia, BR (53); 168- Ituberá, Bahia, BR (53); 169- Canelos, Equador (53); 170- Estacion Biosferica del Beni, Bolívia (53); 171- Ribeirão do Largo, Bahia, BR (53); 172- Serra Norte, Carajás, Pará, BR (53); 173- Entre Rios, Bolívia (53); 174- Ivirgarzama, Bolívia (53); 175- Entre Rios, Bolívia (53); 176- Bolívia (55); 177- Villa Turani, Bolívia (55); 178- Colatina, Espírito Santo, BR (53); 179- Conceição da Barra, Espírito Santo, BR (53); 180- Santa Luzia, Bahia, BR (53); 181- Santa Luzia, Bahia, BR (53); 182- Rio Cushime, Equador (53); 183- Periquito, MG, BR (53); 184- Una, Bahia, BR (53); 185- Porto Seguro, Bahia, BR (53); 186- Ibiraçu, Espírito Santo, BR (53); 187- Estação Biológica de Santa Lucia, Santa Teresa, Espírito Santo, BR (53); 188- Maricá, Rio de Janeiro, BR (56); 189- Espírito Santo, BR (53); 190- Guiana (53); 191- Guiana Francesa (53); 192- Ipiaú, Bahia, BR (53); 193- Iquitos, Loreto, Peru (57); 194- Itabuna, Bahia, BR (53); 195- Itamaraju, Bahia, BR (53); 196- Jacarepaguá, Rio de Janeiro, BR (53); 197- La Chorrera, Colômbia (53); 198- Puerto Arica, Colômbia (53); 199- Apolo, Bolívia (53); 200- Ixiamas, Bolívia (53); 201- Leticia, Colômbia (53); 202- Amacayacu Natural National Park, Leticia, Colômbia (53); 203- Afuá, Pará, BR (53); 204- Marabá, Pará, BR (53); 205- Igrapiúna, Bahia, BR (53); 206- Cotriguaçu, Mato Grosso, BR (53); 207- Filadelfia, Miguelito, Bolívia (53); 208- Filadelfia, Chivé, Bolívia (53); 209- Estacion Biologica Tahuamanu, Bolpebra, Bolívia (53); 210- Palcazu, Chuchurras, Peru (53); 211- Montalvo, Andoas, Equador (53); 212- Kabalebo, Suriname (53); 213- Reserva Florestal de Linhares, Linhares, Espírito Santo, BR (53); 214-Kabalebo, Suriname (53); 215- Santa Cecilia, Equador (53); 216- Alto Alegre, Roraima, BR (53); 217-Tabatinga, Amazonas, BR (53); 218- Tambopata, Peru (53); 219- Teófilo Otoni, Minas Gerais, BR (53); 220- Maués, Amazonas, BR (53); 221- Alenquer, Pará, BR (53); 222- Rio Demerara, Guiana (53); 223- Belterra, Pará, BR (53); 224- Bella Flor, Bolívia (53); 225- Villa Tunari, Bolívia (53); 226- Coca, Equador (53); 227- Costa Marques, Rondônia, BR (53); 228- Espigão do Oeste, Rondônia, BR (53); 229- Rio Essequibo, Guiana (53); 230- Puerto Misahualli, Equador (53); 231- Firmino Alves, Bahia, BR (53); 232- Puerto Galilea, Peru (53); 233- Ibirapitanga, Bahia, BR (53); 234- Iguaí, Bahia, BR (53); 235- João Neiva, Espírito Santo, BR (53); 236- Juína, Mato Grosso, BR (58); 237- Jussari, Bahia, BR (53); 238- Rio Aguarico, Nueva Loja, Equador (53); 239- Limoncocha, Equador (53); 240- Tapanahony, Suriname (53); 241- Leticia, Colômbia (53); 242- Leticia, Colômbia (53); 243- Leticia, Colômbia (53); 244- Domingues, Maranhão, BR (53); 245- Macuma, Equador (53); 246- Madre de Dios, Peru (53); 247- Manaus, Amazonas, BR (53); 248- Mishana, Loreto, Peru (53); 249- Vaupés, Colômbia (53); 250- Nova Mamoré, Rondônia, BR (58); 251- Echarate, Peru (53); 252- Pucallpa, Peru (53); 253- Puerto Maldonado, Madre de Dios, Peru (53); 254- Rio Corrientes, Equador (53); 255- Rio Preto da Eva, Manaus, AM, BR (58); 256- Santa Leopodina, Espírito Santo, BR (59); 257- Santa Maria do Pará, Pará, BR (53); 258- Santa Maria do Salto, Minas Gerais, BR (53); 259- Tambopata, Peru (53); 260- Fitzcarrald, Peru (58); 261- Campoverde, Peru (58); 262- Nauta, Peru (58); 263- Coeroeni, Suriname (58); 264- Yacapana, Venezuela (53); 265- Saint-Elie, French Guiana (58); 266- Sarakreek, Suriname (58); 267- Kabalebo, Suriname (58); 268- Saint-Elie, French Guiana (58); 269- Georgetown, Guyana (53).

# Supplementary References

1. Torrez PPQ, Said R, Quiroga MMM, Duarte MR, França FOS. Forest pit viper (*Bothriopsis bilineata bilineata*) bite in the Brazilian Amazon with acute kidney injury and persistent thrombocytopenia. *Toxicon* (2014) 85:27–30. doi:10.1016/j.toxicon.2014.04.001

2. Dias LG, Feio RN, Santos PS. New record of *Bothriopsis bilineata* (Wied, 1825) (Serpentes, Viperidae) in the Atlantic Forest of Minas Gerais, with a discussion on its conservation. *Lundiana: International Journal of Biodiversity* (2008) 9:75–76. doi:10.35699/2675-5327.2008.23218

3. Cunha OR da. Ofídios da Amazônia I - A ocorrência de *Bothrops bilineatus bilineatus* (Wied) nas matas dos arredores de Belém, Pará (Ophidia, Crotalidae). *Boletim do Museu Paraense Emílio Goeldi Nova série zoologia* (1967) 66:1–12.

4. Debien IV, Waldez F, Menin M. Diversity of reptiles in flooded and unflooded forests of the Amanã Sustainable Development Reserve, central Amazonia. *Herpetology Notes* (2019) 12:1051–1065.

5. Waldez F, Menin M, Vogt RC. Diversity of amphibians and Squamata reptilians from lower Purus River Basin, Central Amazonia, Brazil. *Biota Neotrop* (2013) 13:300–316. doi:10.1590/S1676-06032013000100029

6. Dubeux MJM, Vilela HALS, Andrade AB, Bernarde PS. *Bothrops bilineatus bilineatus* (Two-striped Forest Pitviper): Habitat use. *Herpetological Review* (2019) 50:385–385.

7. Hernández-Ruz EJ, De Olivera EA. New Distributional Records of the Bothrops bilineatus bilineatus (Wied 1825) (Serpentes:Viperidae) in Low Xingu River, Pará, Brazil. *Int J Res Stud Biosci* (2017) 5:1–3.

8. Souza RCG. A Rare Accident. *Bull Chicago Herp Soc* (2007) 42:161–163.

9. Sanz L, Quesada-Bernat S, Pérez A, De Morais-Zani K, SantˈAnna SS, Hatakeyama DM, Tasima LJ, De Souza MB, Kayano AM, Zavaleta A, et al. Danger in the Canopy. Comparative Proteomics and Bioactivities of the Venoms of the South American Palm Pit Viper *Bothrops bilineatus* Subspecies *bilineatus* and *smaragdinus* and Antivenomics of *B. b. bilineatus* (Rondônia) Venom against the Brazilian Pentabothropic Antivenom. *J Proteome Res* (2020) 19:3518–3532. doi:10.1021/acs.jproteome.0c00337

10. Turci LCB, Bernarde PS. Levantamento herpetofaunístico em uma localidade no município de Cacoal, Rondônia, Brasil. *Bioikos, Campinas* (2008) 22:101–108.

11. Souza-Costa CA, Mira-Mendes CVD, Iuri Ribeiro D, Kaique Brito S, Argôlo AJS, Solé M. Squamate reptiles from seasonal semi-deciduous forest remnantsin southwestern Bahia, Brazil. *Bonn Zoological Bulletin* (2020) 69:85–94. doi:10.20363/BZB-2020.69.1.085

12. Moraes L, Almeida A, de Fraga R, Zamora R, Pirani R, Silva A, Carvalho V, Gordo M, Werneck F. Integrative overview of the herpetofauna from Serra da Mocidade, a granitic mountain range in northern Brazil. *ZK* (2017) 715:103–159. doi:10.3897/zookeys.715.20288

13. Frota JG, Santos-Jr AP, Menezes-Chalkidis H, Guedes AG. As serpentes da região do baixo rio Amazonas, oeste do estado do Pará, Brasil (Squamata). *Biociências* (2005) 13:211–220.

14. França RC, Morais M, França FGR, Rödder D, Solé M. Snakes of the Pernambuco Endemism Center, Brazil: diversity, natural history and conservation. *ZK* (2020) 1002:115–158. doi:10.3897/zookeys.1002.50997

15. Silva-Soares T, Ferreira RB, Salles R de OL, Rocha CFD. Continental, insular and coastal marine reptiles from the municipality of Vitória, state of Espírito Santo, southeastern Brazil. *Check List* (2016) 7:290–298. doi:https://doi.org/10.15560/7.3.290

16. Almeida BJL, Almeida MSM, Cavalcante CS, Bernarde PS. *Bothrops bilineatus bilineatus* (Two-striped Forest Pitviper) Reproduction. *Herpetological Review* (2019) 50:385–386.

17. Bernarde PS, Costa HC, Machado RA, São-Pedro VA. *Bothriopsis bilineata bilineata* (Wied, 1821) (Serpentes: Viperidae): new records in the states of Amazonas, Mato Grosso and Rondônia, northern Brazil. *Check List* (2011) 7:343–347.

18. Dal Vechio F, Prates I, Grazziotin FG, Zaher H, Rodrigues MT. Phylogeography and historical demography of the arboreal pit viper *Bothrops bilineatus* (Serpentes, Crotalinae) reveal multiple connections between Amazonian and Atlantic rain forests. *Journal of Biogeography* (2018) 45:2415–2426. doi:10.1111/jbi.13421

19. Argôlo AJS. *As serpentes dos cacauais do Sudeste da Bahia*. Ilhéus, Bahia, Brazil: Editora da UESC (2004).

20. Rojas-Padilla O, Menezes VQ, Dias IR, Argôlo AJS, Solé M, Orrico VGD. Amphibians and reptiles of Parque Nacional da Serra das Lontras: an important center of endemism within the Atlantic Forest in southern Bahia, Brazil. *ZK* (2020) 1002:159–185. doi:10.3897/zookeys.1002.53988

21. Feio RN, Caramaschi U. Contribuição ao conhecimento da herpetofauna do nordeste do estado de Minas Gerais, Brasil. *Phyllomedusa* (2002) 1:105–111.

22. Costa-Campos CE, Sanches PR, Pedroso-Santos F, Figueiredo VAMB, Tavares-Pinheiro R, Almeida-Santos W. Checklist of the reptiles from the Cancão Municipal Natural Park, state of Amapá, eastern Amazon, Brazil. *Herpetology Notes* (2021) 14:539–550.

23. Freitas M. Squamate reptiles of the Atlantic Forest of northern Bahia, Brazil. *Check List* (2014) 10:1020–1030. doi:10.15560/10.5.1020

24. Freitas MA de, Abegg AD, Silva TFS, Fonseca PM, Hamdan B, Filadelfo T. Herpetofauna of Serra do Timbó, an Atlantic Forest remnant in the State of Bahia, Northeastern Brazil. *Herpetology Notes* (2019) 12:245–260.

25. Hamdan B, Silva R. The snakes of Bahia State, northeastern Brazil: species richness, composition and biogeographical notes. *Salamandra* (2012) 48:31–50.

26. Dias IR, Mira-Mendes CV, Solé M. Rapid inventory of herpetofauna at the APA (Environmental Protection Area) of the Lagoa Encantada and Rio Almada, Southern Bahia, Brazil. *Herpetology Notes* (2014) 7:627–637.

27. Avila-Pires TCS, Vitt LJ, Sartorius SS, Zan PA. Squamata (Reptilia) from four sites in southern Amazonia, with a biogeographic analysis of Amazonian lizards. *Boletim do Museu Paraense Emílio Goeldi - Ciências Naturais* (2009) 4:99–118.

28. MacCulloch RD, Reynolds RP. Amphibians and Reptiles from Paramakatoi and Kato, Guyana. *Check List* (2012) 8:207–210.

29. Cole CJ, Townsend CR, Reynolds RP, MacCulloch RD, Lathrop A. Amphibians and reptiles of Guyana, South America: illustrated keys, annotated species accounts, and a biogeographic synopsis. *Proceedings of the Biological Society of Washington* (2013) 125:317–578. doi:10.2988/0006-324X-125.4.317

30. Rivas GA, Molina CR, Ugueto GN, Barros TR, Barrio-Amorós CL, Kok PJR. Reptiles of Venezuela: an updated and commented checklist. *Zootaxa* (2012) 3211:1. doi:10.11646/zootaxa.3211.1.1

31. Jairam R. Checklist of the amphibians and reptiles of the Lely Mountains, eastern Suriname. *Amphibian & Reptile Conservation* (2019) 13:160–171.

32. Ouboter PE, Jairam R, Kasanpawiro C. “A rapid assessment of the amphibians and reptiles of the Kwamalasamutu region (Kutari/lower Sipaliwini Rivers), Suriname,” in *RAP Bulletin of Biological Assessment* A Rapid Biological Assessment of the Kwamalasamutu region, Southwestern Suriname. (Conservation International, Arlington, VA.: O’Shea, B.J., Alonso, L.E., Larsen, T.H.), 124–130.

33. Bernarde PS, Albuquerque S de, Barros TO, Turci LCB. Snakes of Rondônia State, Brazil. *Biota Neotropica* (2012) 12: Available at: http://www.biotaneotropica.org.br/v12n3/en/abstract?inventory+bn00412032012

34. Pantoja DL, Fraga R de. Herpetofauna of the Reserva Extrativista do Rio Gregório, Juruá Basin, southwest Amazonia, Brazil. *cl* (2012) 8:360. doi:10.15560/8.3.360

35. Frazão L, Oliveira ME, Menin M, Campos J, Almeida A, Kaefer IL, Hrbek T. Species richness and composition of snake assemblages in poorly accessible areas in the Brazilian Amazonia. *Biota Neotrop* (2020) 20:e20180661. doi:10.1590/1676-0611-bn-2018-0661

36. da Silva Jr N. The snakes from Samuel hydroelectric power plant and vicinity, Rondônia, Brazil. *Herpetological Natural History* (1993) 1:37–86.

37. Silva MV da, Souza MB de, Bernarde PS. Riqueza e dieta de serpentes do Estado do Acre, Brasil. *Revista Brasileira de Zoociências* (2010) 12: Available at: https://periodicos.ufjf.br/index.php/zoociencias/article/view/24460 [Accessed September 15, 2021]

38. Venegas PJ, Chávez-Arribasplata JC, Almora E, Grilli P, Duran V. New observations on diet of the South American two-striped forest-pitviper *Bothrops bilineatus smaragdinus* (Hoge, 1966). *Cuad Herpetol* (2019) 33: doi:10.31017/CdH.2019.(2017-031)

39. Turci LCB, Albuquerque S de, Bernarde PS, Miranda DB. Uso do hábitat, atividade e comportamento de *Bothriopsis bilineatus* e de *Bothrops atrox* (Serpentes: Viperidae) na floresta do Rio Moa, Acre, Brasil. *Biota Neotrop* (2009) 9:197–206. doi:10.1590/S1676-06032009000300020

40. Grego KF, Fernandes W, Croce AP, Vasconcellos DR, Sant’Anna SS, Coragem JT. *Bothriopsis bilineatus smaragdinus* (Green Jararaca) Reproduction. *Herpetological Review* (2012) 43:492.

41. Hoge AR. Preliminary account on neotropical Crotalinae (Serpentea viperidae). *Memorias do Instituto Butantan* (1965) 32:109–84.

42. Fonseca WL da, Correa RR, Oliveira A de S, Oliveira IS de, Bernarde PS. Habitat use and activity of *Bothrops bilineatus smaragdinus* Hoge, 1966 in the western Brazilian Amazon (Serpentes: Viperidae). *Herpetology Notes* (2021) 14:567–580.

43. Bernarde PS, Albuquerque S de, Miranda DB, Turci LCB. Herpetofauna da floresta do baixo rio Moa em Cruzeiro do Sul, Acre - Brasil. *Biota Neotropica* (2013) 13: Available at: https://www.biotaneotropica.org.br/v13n1/pt/abstract?inventory+bn02113012013

44. Freitas MA de, Venâncio NM, Abegg AD, Azevedo W dos S, Pereira V de O, Zanotti AP, Veloso A, Schwarzbach L, Sousa AGO e, Silva RCC da, et al. Herpetofauna at the Rio Acre Ecological Station, Amazon Rainforest, Brazil. *Herpetology Notes* (2020) 13:33–48.

45. Catenazzi A, Lehr E, Von May. Los anfibios y reptiles del Parque Nacional del Manu y su área de amortiguamiento, cuenca Amazónica y vertientes orientales de los Andes, Perú. *Biota Neotropica* (2013) 13: Available at: https://www.biotaneotropica.org.br/v13n4/pt/abstract?inventory+bn02813042013

46. Metcalf MF, Marsh A, Pacaya ET, Graham D, Gunnels IV CW. Herpetofauna of the Santa Cruz Forest Reserve in the Peruvian Amazon Basin. *Herpetology Notes* (2020) 13:753–767.

47. Cisneros-Heredia DF. Herpetofauna de la Estación de Biodiversidad Tiputini, Amazonía Ecuatoriana: Ecología de una comunidad taxonómicamente diversa, con comentarios sobre metodologías de inventario. in (Universidad San Francisco de Quito. Quito: De la Torre, S. & Reck, G.). doi:10.13140/RG.2.1.2340.5607

48. Doan TM, Arriaga WA. Microgeographic variation in species composition of the herpetofaunal communities of Tambopata Region, Peru. *Biotropica* (2002) 34:101–117.

49. Harvey MB, E JA, A LG. Revision of the venomous snakes of Bolivia. II: The pit viper (Serpentes: Viperidae). *cara* (2005) 74:1–37. doi:10.2992/0097-4463(2005)74[1:ROTVSO]2.0.CO;2

50. Whitworth A, Beirne C. *Reptiles of the Yachana Reserve*. Global Vision International, Exeter (2011).

51. Llanqui IB, Y. Salas C, Oblitas MP. A preliminary checklist of amphibians and reptiles from the vicinity of La Nube Biological Station, Bahuaja-Sonene National Park, Peru. *CheckList* (2019) 15:773–796. doi:10.15560/15.5.773

52. Roberto IJ, Oliveira CR de, Araújo Filho JA de, Oliveira HF de, Ávila RW. The herpetofauna of the Serra do Urubu mountain range: a key biodiversity area for conservation in the brazilian atlantic forest. *Pap Avulsos de Zool (São Paulo)* (2017) 57:347. doi:10.11606/0031-1049.2017.57.27

53. Nogueira CC, Argôlo AJS, Arzamendia V, Azevedo JA, Barbo FE, Bérnils RS, Bolochio BE, Borges-Martins M, Brasil-Godinho M, Braz H, et al. Atlas of Brazilian Snakes: Verified Point-Locality Maps to Mitigate the Wallacean Shortfall in a Megadiverse Snake Fauna. *South American Journal of Herpetology* (2019) 14:1–274. doi:10.2994/SAJH-D-19-00120.1

54. Avila-Pires TCS. “Reptiles,” in *Checklist ofthe terrestrial vertebrates ofthe Guiana Shield* Bulletin of the Biological Society of Washington. (T. HOLLOWELL & R. E. REYNOLDS), 25–40.

55. Fugler CM, De La Riva I, Cabot J. Herpetologica boliviana: una lista comentada de las serpientes de Bolivia con datos sobre su distribucion. *Ecologıa en Bolivia* (1995) 24:40–87.

56. Machado O. Estudos comparativos sôbre ofídios do Brasil. *Boletim do Instituto Vital Brazil* (1945) 5:17–36.

57. Greene HW, Campbell JA. Notes on the use of caudal lures in arboreal green pit vipers. *Herpetologica* (1972) 28:32–34.

58. Campbell JA, Lamar WW. *The Venomous Reptiles of the Western Hemisphere*. New York, USA: Cornell Univ. Press, Ithaca (2004).

59. Ihering R. As cobras do Brazil: Primeira parte. *Revista do Museu Paulista* (1911) 8:273–379.
